# Perceptual Disturbances and Disorders in the ICD-11: An Overview and a Proposal for Systematic Classification

**DOI:** 10.3390/brainsci15010081

**Published:** 2025-01-17

**Authors:** Berthold Langguth, Michael Landgrebe, Dirk De Ridder

**Affiliations:** 1 Department of Psychiatry and Psychotherapy, Bezirksklinikum, University of Regensburg, 93053 Regensburg, Germany; michael.landgrebe@kbo.de; 2 kbo-Lech-Mangfall-Kliniken Agatharied, 83734 Hausham, Germany; 3 Department of Surgery, Section of Neurosurgery, University of Otago, Dunedin 9016, New Zealand; dirk.deridder@otago.ac.nz

**Keywords:** International Classification of Diseases, ICD-11, sensory, tinnitus, pain, vertigo, hallucination, perception, classification

## Abstract

The International Classification of Diseases (ICD) has been developed and edited by the World Health Organisation and represents the global standard for recording health information and causes of death. The ICD-11 is the eleventh revision and came into effect on 1 January 2022. Perceptual disturbances refer to abnormalities in the way sensory information is interpreted by the brain, leading to distortions in the perception of reality. These can manifest as distorted perceptions or as phantom perceptions and can occur in all sensory modalities as visual, auditory, olfactory, gustatory tactile, vestibular, proprioceptory or interoceptory disturbances. There are similar brain mechanisms involved in the generation of these analogous perceptual disturbances and disorders, and they are treated with similar approaches. Perceptual disturbances are highly prevalent, with large variations across the different sensory modalities. They can be associated with significant suffering and cause a high socioeconomic burden. Perceptual disturbances can be symptoms of another disease or disease entities on their own. In the context of pain, this is reflected by the distinction between secondary pain (pain as a symptom of another underlying condition) and primary pain (a disease in its own right, rather than being a symptom of another underlying condition) in the ICD-11. Such a clear distinction is not found in an entirely consistent way across the various sensory modalities. By using the example of auditory phantom perceptions, we propose a framework for the classification of sensory disorders in alignment with the classification of pain in the ICD-11. The descriptions of the sensory disturbances should include (1) a causal aspect (primary versus secondary), (2) a temporal aspect (acute vs. chronic and persistent vs. intermittent), (3) a cognitive, emotional and autonomic interpretation aspect (=suffering) and (4) a social aspect (=disability). If the latter two aspects are present, we propose that the sensory disturbance is called a sensory disorder.

## 1. Introduction

In this perspective article, we discuss the current diagnostic classification of perceptual disturbances in the ICD-11. The focus is not on sensory deficits, but on distorted and phantom perceptions.

The article will not provide a detailed review of similarities and differences in the aetiology or pathophysiology of such perceptual disorders across various modalities, even though this is a research field of great interest. Here, we focus on their current diagnostic classification in the ICD-11. We begin with a brief overview of the structure of the ICD-11, focusing on the classification of disturbances with both somatic and psychological components. We then discuss how perceptual disturbances can be symptoms of another pathology, but also diseases in their own right. The next section provides an overview of the clinical characteristics and current classification of perceptual disturbances in the different sensory modalities.

Finally, using auditory phantom sensations as an example, we propose a framework for the classification of sensory disturbances and disorders along the lines of the classification of pain, and discuss the implications for their inclusion in diagnostic classification systems.

## 2. ICD-11

The International Classification of Diseases (ICD) has been developed by the World Health Organisation as a global standard for the classification of health care entities and causes of death. The ICD-11 is the eleventh revision and came into effect on 1 January 2022. Health care entities include diseases or pathogens, but also isolated symptoms or developmental anomalies of the body. Moreover, the ICD-11 also encompasses reasons for contact with health services, the social circumstances of the patient and external causes of injury or death. An overview over the ICD-11 structure is given in Table 1.

## 3. Overcoming the Mind–Body Dichotomy in the ICD-11

In the ICD-10, categorisation was based on the Cartesian division between ‘organic’ (physical) and ‘non-organic’ (mental) conditions. The ICD-11 aims to take a more holistic approach to health than previous versions, particularly in overcoming the mind–body divide. To this end, efforts have been made to integrate the classification of mental and physical health conditions, reflecting the interaction between mental and physical health rather than treating them as separate entities. This is reflected in the introduction of new chapters covering conditions at the interface between physical and mental health, such as ‘Sexual health’ and ‘Sleep-wake disorders’, which highlight the interrelationship between physical symptoms and psychological conditions: In the ICD-10, sexual dysfunctions that were considered non-organic were included in the chapter on mental disorders, while those that were considered organic were mostly listed in the chapter on “Diseases of the genitourinary system”. The ICD-11 treats the brain and body monistically as an integrated whole, acknowledging the contribution of both physical and psychological factors to sexual dysfunction. Thus, with the introduction of the new chapter “Sexual Health”, the organic versus non-organic distinction has been abolished [1]. Similarly, in the ICD-10, it has been differentiated between sleep disorders (G47), included in the nervous system chapter, and non-organic sleep disorders (F51), included in the mental disorders chapter. In the new “Sleep-wake disorders” chapter in the ICD-11, the separation between organic (physical) and non-organic (mental) disorders has been avoided [2].

The ICD-11 also emphasises the role of psychological and behavioural factors in physical conditions. Chapter 6 on “Mental, Behavioural or Neurodevelopmental Disorders” addresses mental and behavioural disorders that have significant overlap with physical health conditions. It covers a range of disorders, including anxiety and stress-related disorders, which can manifest physically. For example, chapter 6 includes categories for diseases with psychological components that affect physical conditions, such as ‘Somatic symptom disorder’, termed ‘bodily distress disorder’ (6C20).

The ICD-11 also adopts a biopsychosocial model, which considers biological, psychological and social factors in the classification and treatment of diseases. For example, in chronic primary pain, the ICD-11 states that “*Chronic primary pain is multifactorial: biological, psychological and social factors contribute to the pain syndrome*”. Descriptions of disorders in the ICD-11 often include psychosocial factors, encouraging clinicians to consider a wide range of influences on health beyond the purely biological or psychological. This is coded in chapter 6 as “Psychological or behavioural factors affecting disorders or diseases classified elsewhere” (6E40). The ICD-11 also includes a category for functional disorders, also in chapter 6, termed “Dissociative neurological symptom disorder” (6B60), where symptoms cannot be fully explained by structural or biochemical abnormalities. It is described as “*involuntary discontinuity in the normal integration of motor, sensory, or cognitive functions and not consistent with a recognised disease of the nervous system, other mental or behavioural disorder, or other medical condition*”. As mentioned, pain disorders are classified in a way that recognises both physical and psychological components, such as ‘chronic primary pain’, which includes pain without clear physical findings but with significant psychological impact. Trauma- and stress-related disorders are included to cover conditions in which stress and trauma play a central role, reflecting the understanding that psychological stress can have profound physical effects. There is a specific category called “Disorders specifically associated with stress”, which includes PTSD (6B40), complex PTSD (6B41), prolonged grief disorder (6B42) and others.

## 4. Perceptual Disturbances: Symptoms or Disease Entities

Perceptual disturbances can be both symptoms of broader underlying conditions and diseases on their own, depending on their context and underlying causes [3]. The ICD-11 provides a flexible framework that allows perceptual disturbances to be classified either as part of a wider clinical picture or as a primary disturbance.

This can be illustrated by the options for the classification of auditory hallucinations. They can be classified as a symptom of schizophrenia (6A20) as in chapter 6, “Mental, behavioural or neurodevelopmental disorders”, or they can be classified per se as MB27.20 under “Symptoms or signs involving perceptual disturbance” (MB27) as in chapter 21, “Symptoms, signs or clinical findings, not elsewhere classified”.

## 5. Classification of Perceptual Disorders in the ICD-11

Most perceptual disturbances fall under chapter 21, *Symptoms, signs or clinical findings, not elsewhere classified*, under the heading *Mental or behavioural symptoms, signs or clinical findings*, and subheading MB27: *Symptoms or signs involving perceptual disturbance.* This is described as “Symptoms and signs involving a disruption in sensory perception, including depersonalization, derealization and hallucinations in any modality”. Hallucinations (MB27.2) are described as “Sensory perceptions of any modality occurring in the absence of the appropriate (external) stimulus. The person may or may not have insight into the unreal nature of the perception.” Hallucinations are differentiated from illusions (MB27.4), which are described as “A misinterpretation of a true sensation (e.g., hearing voices in the sound of running water, the perception of figures in shadows)”.

Pain, however, also in chapter 21, falls under a different subheading: *General symptoms, signs or clinical findings*.

### 5.1. Visual

Visual hallucinations and illusions can occur as symptoms of various disorders. They are a hallmark of migraine aura (8A80.1 *Migraine with Aura*) but also a frequent symptom in delirant states (6D70), e.g., in the context of alcohol withdrawal syndrome (6C40). They can also occur as scenic hallucinations in psychotic states caused by the intake of psychoactive substances (6C4*) or as a symptom of schizophrenia (6A20).

Visual hallucinations are coded as MB 27.27 and defined as follows: “Hallucinations involving sight in the absence of an actual visual stimulus that are not restricted to the period of awakening or the onset of sleep. Visual hallucinations may involve formed images, such as of people, or of unformed images, such as flashes of light. Visual hallucinations must be distinguished from illusions, which are visual misperceptions of real external stimuli”. Visual illusions (9D54) are excluded from MB27.4 and are separately defined as “percepts based on an erroneous interpretation of visual input”. This is described in chapter 9, “Diseases of the visual system”. Thus, whereas hallucinations are coded under chapter 21, illusions fall under the visual system. Similarly, visual phantom perceptions occurring in the visually impaired in the absence of any psychiatric/neurological disorder, also known as Charles Bonnet syndrome, are coded in chapter 9 as “Visual release hallucinations” (9D56). In other words, like illusions, they are classified under chapter 9, *Diseases of the visual system*, under the heading *Impairment of visual functions*, and subheading *Subjective visual experiences*, separate from but alongside visual illusions: “Visual release hallucinations, also called Charles Bonnet syndrome, refer to the experience of complex visual hallucinations in a person who has experienced partial or complete loss of vision. Hallucinations are exclusively visual, usually temporary, and unrelated to mental and behavioural disorders”.

Another visual phantom perception is visual snow, which is characterised by the perception of constant, innumerable flickering dots throughout the visual field and for which no specific code is available in the ICD-11. Among patients experiencing visual snow syndrome, there is an increased prevalence of somatosensory (pain) dysfunction such as migraine and fibromyalgia, auditory dysfunction such as tinnitus, and vestibular dysfunction such as dizziness [4,5]. These comorbidities suggest an overlap in the pathophysiological mechanisms of perceptual disorders in the various sensory modalities.

In summary, visual perceptual disorders can be coded as visual hallucinations or visual illusions per se or as symptoms of underlying diseases. The amount of suffering that is related to visual perceptual disturbances is not part of the definitions.

### 5.2. Auditory

Auditory hallucinations (MB27.20) in chapter 21 are defined as “Hallucinations involving the perception of sound, most frequently of voices but sometimes of clicks or other noises that are not restricted to the period of awakening or the onset of sleep”. This definition includes auditory hallucinations in the form of voices, which typically occur in the context of schizophrenia (6A20), musical hallucinations (not classified), which occur in the context of profound hearing loss [6], and tinnitus, which typically manifests as a tonal or noise-like sound (MC41).

Tinnitus, in turn, is defined as “A nonspecific symptom of hearing disorder characterised by the sensation of buzzing, ringing, clicking, pulsations and other noises in the ear in the absence of appropriate corresponding external stimuli and in the absence of what the examiner can hear with a stethoscope”.

Hyperacusis, which is characterised by increased sensitivity to sound and by a low tolerance for environmental noise, is classified under “*Other specified disorders of ear, not elsewhere classified*” (AB7Y). This is a subheading of chapter 10: “Diseases of the ear or mastoid process”.

Phonophobia, which is typically defined as a persistent, abnormal and unwarranted fear of sound [7], is listed in the ICD-11 under MB42 in chapter 21 and defined as “*Hypersensitivity to sounds*”, which corresponds more to the clinical description of hyperacusis.

Misophonia, a disorder that is characterised by decreased tolerance to specific sounds or their associated stimuli, is not listed in the ICD-11.

Analogous to visual perceptual disturbances, the amount of suffering that is related to the various auditory perceptual disorders is not part of the definitions.

In summary, some auditory perceptual disturbances are coded under chapter 10, and considered ear disease-associated pathologies, and others are coded under chapter 21: *Symptoms, signs or clinical findings, not elsewhere classified*.

### 5.3. Somatosensory

Most somatosensory perceptual symptoms and syndromes are some form of pain or related to pain. From a physiological perspective, there exist two somatosensory systems, the lemniscal system and the neospinothalamic system. These two systems contribute together to cutaneous, musculoskeletal and visceral sensory perceptions. Pain sensations (protopathic and epicritic) are formed on the basis of both the lemniscal system and the neospinothalamic somatosensory systems.

However, pain may or may not be a symptom of a perceptual disorder. If pain is a symptom of another condition, such as trauma or inflammation, it is not a perceptual disturbance, but a physiological response to structural damage, transmitted via a dedicated pain pathway. In contrast to the visual, auditory, vestibular and olfactory sensory systems, there exists a specific pain-transmitting pathway in the nervous system. There exists no tinnitus pathway, nor vertigo pathway, nor visual snow pathway.

The ICD-11 codes mostly pain in chapter 21, but pain disorder (8E43) in chapter 8: *Diseases of the nervous system*. Pain disorder in ICD-11 equals neuropathic pain (8E43.0), but it excludes chronic neuropathic pain (MG30.5), which is coded in chapter 21, with the other pain types. In chapter 21, the ICD-11 differentiates between primary pain (where pain itself is a disease) and secondary pain (where pain is a symptom of another disease). Moreover, it differentiates between acute pain (pain with a duration of less than 3 months) and chronic pain (duration of more than 3 months). Acute pain is classified according to the pain origin (face, head, postoperative, specified, or unspecified). Chronic secondary pain is also classified according to its region or origin, e.g., visceral, musculoskeletal, or cancer. A specific form of secondary pain is chronic neuropathic pain (8E43.0), which is described as “chronic pain caused by a lesion or disease of the somatosensory nervous system”.

Chronic primary pain (MG30 in chapter 21: *Symptoms, signs or clinical findings, not elsewhere classified*) is described as “chronic pain in one or more anatomical regions that is characterised by significant emotional distress (anxiety, anger/frustration or depressed mood) or functional disability (interference in daily life activities and reduced participation in social roles)”. For a more granular description, extension codes (chapter X) can be used, e.g., for coding pain severity (no, mild, moderate, severe), pain distress (no, mild, moderate, severe), pain interference (no, mild, moderate, severe) and the time course (e.g., intermittent, persistent).

Moreover, specific sensory disturbances, including allodynia (MB40.1; pain due to a normally non-painful stimulus), hyperaesthesia (MB40.5; increased sensibility to stimuli of sense), or dysaethesia (MB40.6; unpleasant, abnormal sense of touch), are listed in MB40 (Sensation Disturbances, chapter 21).

Diagnostic categories in the somatosensory modality differ from the other modalities, as there is a differentiation between primary and secondary disorders, and acute and chronic disorders, and a grading depending on severity, distress and behavioural impairment. Moreover, the definition of chronic primary pain refers to a biopsychosocial disease model.

### 5.4. Vestibular

Perceptual disturbances in the vestibular modality can be classified under MB48,00 in chapter 21, which is defined as “an abnormal processing of the vestibular sensory input by the central nervous system due to either disruption of central integrators in the brain stem or cerebellum (=organic) or sensory information mismatch from the cortex (=functional)”. Vestibular dysfunction is, however, classified as Persistent Postural-Perceptual Dizziness (AB32.0) in chapter 10, *Diseases of the ear or mastoid process*, under code AB32, Chronic vestibular syndrome, which includes phobic postural vertigo and which is defined as “persistent non-vertiginous dizziness and/or unsteadiness, lasting three months or more. Symptoms are present most days, often increasing throughout the day, but may wax and wane. Momentary flares may occur spontaneously or with sudden movement. Affected individuals feel worst when upright, exposed to moving or complex visual stimuli, and during active or passive head motion. These situations may not be equally provocative. Typically, the disorder follows occurrences of acute or episodic vestibular or balance-related problems. Symptoms may begin intermittently, and then consolidate. Gradual onset is uncommon”. Moreover, dizziness can be classified as MB48 under *Sensation Disturbances* (chapter 21).

Mal de debarquement, also known as disembarkement syndrome, which can occur after unfamiliar motion patterns, e.g., after travelling or after an earthquake [8,9], is classified under chapter 10, *Diseases of the ear or mastoid process* (heading: *Diseases of inner ear*; subheading AB31: *Episodic vestibular syndrome)*. Its definition there is as follows: “Disembarkment syndrome, or Mal de debarquement (MdD) occurs when habituation to unfamiliar motion patterns like traveling on a boat, train, or airplane, becomes resistant to re-adaption on return to stable conditions. It results in an illusion of self motion typically described as rocking, bobbing, or swaying. Brief periods of MdD (hours) are common in healthy individuals, this otherwise natural phenomenon can become persistent in some individuals”. Even though it is described as an illusion, it does not fall under illusions (MB27.4) in chapter 21.

Analogous to auditory and visual perceptual disturbances, the amount of suffering that is related to vestibular disorder is not part of the definitions. Also analogous to the auditory system, some perceptual disturbances are coded in the ear in chapter 8, and others in chapter 21.

### 5.5. Gustatory/Olfactory

Gustatory hallucinations (MB27.21) and olfactory hallucinations (MB27.24) are both listed in chapter MB27: *Symptoms or signs involving perceptual disturbance*. Gustatory hallucinations are simply defined as “Hallucinations of taste in the absence of an actual external stimulus”. Olfactory hallucinations are defined as “Hallucinations involving the perception of odour (e.g., of burning rubber, decaying fish, orange peel) in the absence of an actual external stimulus”.

Parosmia (MB41.1), the abnormal perception of smell, and dysgeusia, the altered perception of taste (MB41.2), are both listed in the MB41 of chapter 21: *Disturbances of smell and taste*. Analogous to auditory and visual perceptual disturbances, the amount of suffering that is related to gustatory and olfactory disorder is not part of the definitions.

### 5.6. Hypnagogic and Hypnopompic Hallucinations

Hypnopompic (MB27.22) and hypnagogic (MB27.23) hallucinations are defined as *hallucinations that occur at the onset of sleep (hypnagogic) and during the period of awakening (hypnopompic), respectively, most commonly of the visual, tactile, or auditory modality*. They can both occur in the context of narcolepsy Type 1 (7A20.0).

### 5.7. Hypersensitivity Disturbances (Hypersensitivity to Electromagnetic Fields, Multiple Chemical Sensitivity Syndrome, Sick Building Syndrome)

Hypersensitivity to electromagnetic fields, multiple chemical sensitivity syndrome, or sick building syndrome are all syndromes characterised by a variety of non-specific symptoms that are attributed by sufferers to environmental factors such as electromagnetic fields or chemical substances. However, no clear evidence of such a causal relationship has been found in exposure studies. Anxiety and suggestibility seem to contribute to the development of symptoms. All these so-called Hypersensitivity Syndromes are not coded in the ICD-11. This is of relevance as the inability to code a health entity means that there is no awareness of it in the medical field, or to put it a little more pointedly: “if it can’t be coded, it doesn’t exist”, as suggested by the title of a recent manuscript [10].

## 6. Proposed Framework for Classification of Perceptual Disorders

Comparing the categorization of perceptual disturbances in the various sensory modalities in the ICD11 reveals relevant differences. In the olfactory and gustatory domain, they are just described, ether as abnormal or as hallucinatory perceptions. They are uniquely encoded in chapter 21, titled *Symptoms, signs or clinical findings, not elsewhere classified*. The reason is that no separate chapter exists for the olfactory or gustatory system. Visual, auditory and vestibular perceptual disturbances are either coded in chapters related to the sensory system (chapter 9: visual; chapter 10: audiovestibular) or in chapter 21 (unclassified). For the visual system, specific abnormal perceptions, which occur as symptoms of other disorders (e.g., visual hallucinations in the context of migraine), are specifically characterised, in addition to more general terms such as visual hallucinations. In the vestibular domain, different disorders are differentiated according to their time course (acute, chronic persistent, intermittent) and their origin (central vertigo). Some perceptual changes are coded under the ear or mastoid process chapter (chapter 10) and others in the unclassified chapter (chapter 21). For example, for the vestibular system, *Persistent Postural-Perceptual Dizziness* and *Mal de debarquement* are coded in chapter 10, whereas vertigo is coded in chapter 21. For the auditory system, most perceptual problems are classified in chapter 21, but hyperacusis falls under chapter 10.

The by far most systematic differentiation can be found for the various forms of pain and the somatosensory system. First, whether pain is the symptom of another disorder (secondary pain) or whether pain is a disorder by itself (primary pain) is differentiated. In addition, a distinction is made between acute and chronic pain. Whereas acute pain is typically secondary pain, chronic pain can be either primary or secondary. Further specifications include the body region and the extent of emotional distress or functional disability. Pain severity (no, mild, moderate, severe), pain distress (no, mild, moderate, severe), pain interference (no, mild, moderate, severe) and the time course (e.g., intermittent, persistent) can be characterised by additional extension codes. Even though most pain codes are described in chapter 21, analogous to the other senses, some specific forms of neuropathic pain, such as the phantom pain sensation in the context of phantom limb syndrome, fall under chapter 8, *Diseases of the nervous system*, under the heading *Other disorders of the nervous system*, with subheading 8E43, *Pain disorders*, and code 8E43.0, *Neuropathic pain.* Phantom limb syndrome is described as “… the perception of sensations, including pain, in a limb that has been amputated or a body part that has been removed. These sensations may include a specific position, shape, or movement of the phantom, feelings of warmth or cold, itching, tingling, or electric sensations, and other paraesthesias”. As such, even though phantom pain is a sensory perception in the absence of a corresponding external stimulus, it is not classified as hallucinatory in nature, which would fall under MB27.2.

It may be of use to develop a more systematic approach to describing and defining perceptual disturbances and disorders. One approach could be to start with a physiologically based definition of sensation and perception. Perception has been defined as the act of interpreting and organising a sensory stimulus to produce a meaningful experience of the world and of oneself [11]. A stimulus produces an effect on the different sensory receptors, which is transmitted to the sensory cortex, inducing sensation [11]. Further processing of this sensory stimulation by other brain networks, such as the default mode, salience network and frontoparietal control network, generates an internal representation of the outer and inner world called a percept [11]. Perception can thus be defined as the act of interpreting and organising a sensory stimulus or the absence of a sensory stimulus to produce a meaningful experience of the self in the surrounding world [11]. This would imply, for classifying purposes, that perceptual disorders need to be looked at in four aspects: (1) the causal aspect, which is the sensory percept related to a clear cause (secondary) or not (primary); (2) the temporal aspect (acute versus chronic, intermittent versus persistent); (3) the cognitive, emotional and autonomic aspect, which results in associated suffering or not; and (4) the social aspect, which corresponds to disability. Thus, for classification purposes, the sensation or lack thereof, such as in deafferentation, could be coded in the different sensory chapters (visual (9), auditory vestibular (10)) or the nervous system (chapter 8), or it would require that chapters be added (olfactory, gustatory, somatosensory).

The perceptual aspects, i.e., cognitive, emotional and autonomic, could fall under chapter 21, as could social factors. Yet, creating a homogenous approach to classifying all sensory disorders in the same way may be useful, as pathophysiological and clinical overlap exists between the different sensory systems, and it would make sense to stratify coding based on pathophysiological and clinical aspects.

Therefore, it is tempting to apply the conceptual framework for the classification of the different forms of pain to other sensory modalities. This would require categorization according to the categories in Table 2.

In order to illustrate how these categories could be applied to perceptual disorders in other modalities, we use the example of tinnitus, an auditory phantom percept. The current ICD11 definition describes tinnitus under MC41 as “A nonspecific symptom of hearing disorder characterized by the sensation of buzzing, ringing, clicking, pulsations, and other noises in the ear in the absence of appropriate corresponding external stimuli and in the absence of what the examiner can hear with a stethoscope” [12] within chapter 10: *Symptoms and signs involving the ear*, specifically under *Other specified symptoms and signs involving the ear*. However, this definition has several shortcomings:

1. Tinnitus is not only a symptom (secondary tinnitus), but can also be a self-standing disorder in its own right (primary tinnitus), similar to pain. It can be a secondary symptom of conditions like Meniere’s disease or otosclerosis, but also can arise without an obvious or detectable cause.
  And similarly, as primary pain can be classified in the ICD-11 as its own entity, a corresponding category would be appropriate for primary tinnitus.
2. The definition neglects the affective, cognitive and autonomic components of tinnitus-related distress [13]. Like pain, tinnitus encompasses not only sensory aspects but also emotional distress and functional impairment, including depression, anxiety, sleep disturbances, and cognitive issues [14], resulting in a highly variable impact on quality of life. For assessing tinnitus severity and for guiding clinical interventions, various self-report questionnaires have been developed.
3. Tinnitus can occur in the absence of a clinically detectable hearing disorder, even though cochlear dysfunction may be detectable through advanced tests [15,16,17,18].
4. There are many other kinds of sounds that people with tinnitus hear that fall outside the current ICD11 definition, including sea- or electricity-like noise or crickets or combinations of the abovementioned.
5. This definition excludes what is now still called objective tinnitus or somatosounds, as it defines tinnitus as ‘… *in the absence of what the examiner can hear with a stethoscope*’.
6. The definition lacks time criteria.
7. The definition is not grounded in a neurobiologically inspired pathophysiological understanding of the generation of tinnitus.

Applying the proposed categories (Table 2), one could differentiate between primary and secondary tinnitus, the time course and its severity, which would address the major criticisms of the current definition.

### 6.1. Causal Aspect: Primary Versus Secondary Tinnitus

As mentioned above, tinnitus can be a symptom of conditions like hearing loss, Meniere’s disease or cochlear migraine, but it can arise without an obvious or detectable cause and represent a disease entity on its own. The former would classify as secondary tinnitus, and the latter as primary tinnitus. In this context, the distinction between subjective tinnitus and objective tinnitus also has to be considered. Subjective tinnitus is only perceived by the affected person and is the most common type. Objective tinnitus, also described by the term somatosounds, is a real sound that can be heard by others as well (e.g., by using a stethoscope) and is caused by internal sound sources like vascular abnormalities or muscular contractions. Objective tinnitus would clearly be classified as secondary tinnitus, whereas subjective tinnitus can be either primary or secondary tinnitus.

### 6.2. Temporal Aspect

For the differentiation between acute and chronic tinnitus, typically, a duration of 3–6 months is chosen. There are increasing data from neuroimaging, demonstrating that the pathophysiologic mechanisms differ between tinnitus wit a recent onset and tinnitus with a longer duration, but there is no empirical evidence for a specific cut-off for the transition from acute to chronic tinnitus [19]. Acute tinnitus could be considered a secondary symptom of an underlying problem, such as traumatic or non-traumatic hearing loss, and metabolic, nutritional, infectious, genetic, autoimmune, and/or vascular processes. If it becomes chronic, tinnitus may become a primary disorder in its own right, analogous to the concept of pain [3]. In addition, the temporal aspects of when the auditory phantom sensation turns into a psychological/psychiatric problem, i.e., a disorder, are currently still incompletely understood. This differs among patients, whether the suffering starts simultaneously with the auditory sensation of tinnitus or whether the suffering develops gradually and stabilises after a certain moment in time, and whether once stable it could be considered chronic or persistent and turn into a disorder. Chronic pain was defined previously as pain that persists past normal healing time, and hence, lacks the acute warning function of physiological nociception [3], but in the ICD11, it is now simplified based on a temporal criterion of 3 months [3,20,21].

From a clinical perspective, and analogous to former definitions of chronicity in pain, a differentiation between acute and chronic tinnitus could be guided by the chance of spontaneous recovery or by the efficacy of specific interventions for acute tinnitus, but here, there also exist no convincing data. Given the lack of convincing empirical data it seems appropriate to adopt the 3-month demarcation between “acute” and “chronic” from the pain definition for tinnitus according to the pathophysiological, clinical and treatment analogies between tinnitus and pain [22,23,24,25,26,27]. The differentiation between constant and intermittent is relevant, as intermittent forms of tinnitus differ in their pathophysiology from constant tinnitus. Intermittent tinnitus can occur in the context of specific disorders, such as Meniere’s disease, cochlear migraine or rare forms of epilepsy. Thus, it is highly recommended that a differentiation between constant and intermittent be made in the diagnostic classification of tinnitus.

### 6.3. Cognitive, Emotional and Autonomic Interpretation Aspect and Disability: Sensory Disturbance or Sensory Disorder?

In the diagnostic classification of perceptual disturbances, it is essential to consider the amount of suffering, and its social impact, i.e., the related disability and interference with daily life activities. This is not trivial, as the amount of suffering can vary considerably, ranging from no suffering at all to suicidality. The amount of suffering also determines whether a perceptual disturbance might be considered as a symptom or as a disorder on its own. The majority of people who perceive a phantom sound experience no suffering associated with it. Yet, for some of the persons affected, tinnitus represents an unpleasant experience associated with negative cognitive, emotional and autonomic reactions [28], leading to disability and interference with daily life, and this should be reflected in the diagnostic classification.

For these reasons it has been recently proposed in a consensus paper [19] that tinnitus without and with associated suffering should be differentiated by distinct terms: “Tinnitus” for the former and “Tinnitus Disorder” for the latter. The proposed definition is “*Tinnitus is the conscious awareness of a tonal or composite noise for which there is no identifiable corresponding external acoustic source*”, which becomes Tinnitus Disorder “*when associated with emotional distress, cognitive dysfunction, and/or autonomic arousal, leading to behavioural changes and functional disability*”. In other words, “Tinnitus” describes the auditory or sensory component, whereas “Tinnitus Disorder” reflects the auditory component and the associated suffering. The amount of suffering can then be graded according to the number and severity of the associated affective, cognitive and autonomous symptoms.

## 7. Conclusions

The ICD-11 classification for perceptual disturbances in the various modalities varies considerably. The most sophisticated differentiation exists for the classification of pain. Here, the differentiation is made according to the categories “primary versus secondary” and “time course” (acute versus chronic, intermittent versus persistent) and according to severity (amount of distress, functional disability and interference). We propose using this framework for the classification of perceptual disturbances in all sensory modalities and illustrate the advantages of this approach with the example of tinnitus. In particular we demonstrate the relevance of associated suffering, which is a highly relevant criterion for diagnostic classification and also for categorization as a symptom and a disorder on its own. Therefore, we propose that the descriptions of the sensory disturbances should include (1) a causal aspect (primary versus secondary), (2) a temporal aspect (acute vs. chronic and persistent vs. intermittent), (3) a cognitive, emotional and autonomic interpretation aspect (=suffering) and (4) a social aspect (=disability). If the latter two aspects are present, we propose that the sensory disturbance is called a sensory disorder.

## Figures and Tables

**Table 1:** Structure of the ICD-11 (https://icd.who.int/en (accessed on 15 November 2024)).

	Range	Chapter	#	Range	Chapter
1	1A00–1H0Z	Certain infectious or parasitic diseases	15	FA00–FC0Z	Diseases of the musculoskeletal system or connective tissue
2	2A00–2F9Z	Neoplasms	16	GA00–GC8Z	Diseases of the genitourinary system
3	3A00–3C0Z	Diseases of the blood or blood-forming organs	17	HA00–HA8Z	Conditions related to sexual health
4	4A00–4B4Z	Diseases of the immune system	18	JA00–JB6Z	Pregnancy, childbirth or the puerperium
5	5A00–5D46	Endocrine, nutritional or metabolic diseases	19	KA00–KD5Z	Certain conditions originating in the perinatal period
6	6A00–6E8Z	Mental, behavioural or neurodevelopmental disorders	20	LA00–LD9Z	Developmental anomalies
7	7A00–7B2Z	Sleep-wake disorders	21	MA00–MH2Y	Symptoms, signs or clinical findings, not elsewhere classified
8	8A00–8E7Z	Diseases of the nervous system	22	NA00–NF2Z	Injury, poisoning or certain other consequences of external causes
9	9A00–9E1Z	Diseases of the visual system	23	PA00–PL2Z	External causes of morbidity or mortality
10	AA00–AC0Z	Diseases of the ear or mastoid process	24	QA00–QF4Z	Factors influencing health status or contact with health services
11	BA00–BE2Z	Diseases of the circulatory system	25	RA00–RA26	Codes for special purposes
12	CA00–CB7Z	Diseases of the respiratory system	26	SA00–SJ3Z	Supplementary Chapter Traditional Medicine Conditions—Module I
13	DA00–DE2Z	Diseases of the digestive system	27	VA00–VC50	Supplementary section for functioning assessment
14	EA00–EM0Z	Diseases of the skin	28	XA0060–XY9U	Extension Codes

**Table 2:** Categories.

Causal aspect	Primary versus secondary
Temporal aspect	Acute versus chronic; intermittent versus persistent
Cognitive, emotional and autonomic aspect	Degree of suffering
Social aspect	Degree of disability
